# Differences in Linear Epitopes of Ara h 9 Recognition in Peanut Allergic and Tolerant, Peach Allergic Patients

**DOI:** 10.3389/falgy.2022.896617

**Published:** 2022-07-22

**Authors:** L. Sánchez-Ruano, C. Fernández-Lozano, M. Ferrer, F. Gómez, B. de la Hoz, J. Martínez-Botas, M. J. Goikoetxea

**Affiliations:** ^1^Allergy Department, Hospital Universitario Ramón y Cajal, Instituto Ramón y Cajal de Investigación Sanitaria (IRYCIS), Madrid, Spain; ^2^Servicio de Bioquímica-Investigación, Hospital Universitario Ramón y Cajal - Instituto Ramón y Cajal de Investigación Sanitaria (IRYCIS), Madrid, Spain; ^3^Department of Allergy and Clinical Immunology, Clínica Universidad de Navarra, Navarra Health Research Institute (IDISNA, Instituto de Investigacion Sanitaria de Navarra), Pamplona, Spain; ^4^Research Network on Asthma, Drug Adverse Reactions and Allergy (ARADyAL, Red de Investigacion en Asma, Reacciones Adversas a Farmacos y Alergia), Málaga, Spain; ^5^Allergy Clinical Unit, Hospital Regional Universitario de Málaga, Instituto de Investigación Biomédica de Málaga (IBIMA), Málaga, Spain; ^6^CIBER de Fisiopatología de la Obesidad y Nutrición (CIBEROBN)-Instituto de Salud Carlos III, Madrid, Spain

**Keywords:** peanut, lipid transfer protein, linear epitopes, peach, IgE, IgG4, cross reactivity

## Abstract

**Background:**

Peanut-allergic patients from the Mediterranean region are predominantly sensitized to the lipid transfer protein (LTP) Ara h 9, and the peach LTP Pru p 3 seems to be the primary sensitizer. However, LTP sensitization in peanut allergy is not a predictive marker for clinically relevant symptoms.

**Objective:**

We aimed to identify sequential epitopes of IgE and IgG4 from Pru p 3 and Ara h 9 in peach-allergic patients sensitized to peanuts. We also sought to determine the differences in IgE and IgG4 binding between patients who had developed peanut allergy and those tolerating peanuts.

**Methods:**

A total of 46 peach-allergic patients sensitized to peanuts were selected. A total of 35 patients were allergic to peanuts (peanut-allergic group) and 11 were tolerant to peanuts (peanut-tolerant group). We measured sIgE and sIgG4 in peanut, peach, and their recombinant allergen (Ara h 1, Ara h 2, Ara h 3, Ara h 8, and Ara h 9) with fluorescence enzyme immunoassay. We examined the IgE and IgG4 binding to sequential epitopes using a peptide microarray corresponding to linear sequences of the LTPs Ara h 9 and Pru p 3 with a library of overlapping peptides with a length of 20 amino acids (aa) and an offset of 3 aa.

**Results:**

The frequency and the intensity of IgE recognition of Ara h 9 and Pru p 3 peptides were higher in the peanut-tolerant group than in the peanut-allergic group. We found four Ara h 9 peptides (p4, p14, p21, and p25) and four Pru p 3 peptides (p1, p3, p21, and p24) with a significantly elevated IgE recognition in peanut-tolerant patients. Only one peptide of Ara h 9 (p4) recognized by IgG4 was significantly elevated in the peanut-tolerant group. The IgG4/IgE ratio of Ara h 9 peptide 4 was significantly higher in peanut-tolerant patients than in peanut-allergic patients, while no significant differences were observed in the IgG4/IgE ratio of this peptide in Pru p 3.

**Conclusion:**

Although we found significant differences in IgE and IgG4 recognition of Ara h 9 and Pru p 3 between peanut-tolerant and peanut-allergic patients (all of whom were allergic to peach), polyclonal IgE peptide recognition of both LTPs was observed in peach-allergic patients tolerating peanuts. However, the IgG4 blocking antibodies against Ara h 9 peptide 4 could provide an explanation for the absence of clinical reactivity in peanut-tolerant peach-allergic patients. Further studies are needed to validate the usefulness of IgG4 antibodies against Ara h 9 peptide 4 for peanut allergy diagnosis.

## Introduction

Peanut allergy is one of the most common causative agents of food allergy (1). In the Mediterranean region, peanut-allergic adult patients are mostly sensitized to the lipid transfer protein (LTP) Ara h 9 (2–5). This pattern has also been observed in other non-Mediterranean countries (6).

It has been suggested that the peach LTP Pru p 3 may be the origin of peanut sensitization (2, 5) since co-sensitization to peanut and peach is frequent in LTP-sensitized food-allergic patients (7, 8) and cross-inhibition assays have revealed a strong inhibitory capacity of Pru p 3 against Ara h 9 that is not reproduced inversely (5). Positive specific IgE to both peach and peanut and their LTPs, Pru p 3 and Ara h 9, is frequently observed in LTP-sensitized patients, as is the expression of this sIgE cross-sensitization. However, the clinical expression of these sensitizations is not always observed (2, 5, 9, 10). As such, discriminative clinically relevant markers for peanut allergy in peach-allergic patients are essential for the proper management of these patients in clinical practice.

The aim of this study was to identify linear IgE epitopes of the major allergen in LTP-sensitized peanut-allergic patients, Ara h 9 and its primary sensitizer Pru p 3 for its clinical relevance to peanut sensitization in peach-allergic patients.

## Materials and Methods

### Patients

Subjects were selected from a previous prospective study at the allergology departments of Hospital Carlos Haya in Málaga and Clínica Universidad de Navarra in Pamplona, Spain. This study evaluated LTP-sensitization prevalence and primary sensitization in peanut-sensitized and allergic patients (5) (90%) and in peanut-tolerant patients prior to evaluating sublingual peach immunotherapy (11) (10%). Non-pediatric patients (>14 years old) with an immediate type reaction after eating a peach and a positive skin-prick test (SPT) to this fruit extract in the previous study were enrolled in the current study. All patients showed sensitization by SPT to peanut extract. Patients were assigned to either a peanut-allergic group (presenting sIgE-mediated symptoms upon peanut ingestion within the last 2 years) or a peanut-tolerant group (based on an open food challenge with 14–20 g of roasted peanuts). The study was conducted according to the Declaration of Helsinki, Good Clinical Practice and local regulations. All participants were informed about the study by the medical team and signed an informed consent approved by the ethics committee.

### Skin Tests

Skin prick tests (SPT) were performed with commercial peanut extract [Bial-Aristegui (Bilbao, Spain) or ALK-Abelló (Madrid, Spain)] and LTP-enriched peach extract (30 μg/ml of Pru p 3) (ALK- Abelló). Wheals with a 3-mm diameter were considered positive, as recommended by the European Academy of Allergy and Clinical Immunology (12).

### Oral Food Challenge

Tolerance to peanuts was confirmed by an open oral peanut challenge with 14 to 20 g of roasted peanut in peanut-tolerant patients. The tests were performed following the European Academy of Allergy and Clinical Immunology (EAACI) recommendations (13).

### sIgE and sIgG4 to Peanut, Peach and Their LTP Levels

We measured sIgE to peanut extract and the recombinant (r) allergen rAra h 1, rAra h 2, rAra h 3, rAra h 8, rAra h 9, and sIgE to peach extract and rPru p 3 with fluorescence enzyme immunoassay (FEIA) [ImmunoCAP™ (Thermo Fisher Scientific, Uppsala, Sweden)], according to the manufacturer's instructions. Serum sIgE values were quantified in kilounits of allergen (kUA) per liter, with values of 0.35 kUA/L or greater considered positive.

We measured sIgG4 to rPru p 3 and to rAra h 9 by ImmunoCAP™ (measuring range: 0.07–30 mgA/L. Cut-off points for sIgG4 are not established).

### Peptide Microarray Immunoassay

A library of overlapping peptides with a length of 20 amino acids (aa) with an offset of 3 aa, corresponding to the primary sequences of Ara h 9 and Pru p 3, was commercially synthesized (GenScript Corporation, Piscataway, N.J., USA). Peptides were resuspended in phosphate-buffered saline (PBS) and diluted (1:1) with protein-printing buffer (PPB) (Arrayit Corporation, Sunnyvale, Calif., USA) to a final concentration of 1 mg/ml. All peptide samples were printed on glass slides coated with N-Hydroxylsuccinimidyl (NSB27 NHS; NanoSurface BiosciencesPOSTECH, Seoul, Korea) using a SpotArray™ 72 spotter with SMP6 split pins (PerkinElmer, Waltham, Mass., USA). PBS/PPB (1:1) was used as a negative control and for background normalization. The slides were rinsed with PBS containing 0.1% Tween^®^ 20 detergent (PBS-T), and nonspecific binding sites were blocked for 60 min with BlockIt^TM^ blocking buffer (Arrayit Corporation) diluted 1:1 in PBS-T containing 2% bovine serum albumin (BSA) (Sigma-Aldrich, St. Louis, Mo., USA). The slides were washed 2 times with PBS-T and the patient sera diluted 1:2 in PBS-T 2% BSA was applied and incubated for 14 h on a rotator at 4 C. The slides were then washed 2 times with PBS-T and incubated for 2 h at room temperature with a mixture of monoclonal mouse anti-human IgE (clone G7-26) and IgG4 (clone G14-4) (BD-Pharmingen, San Diego, Calif., USA) diluted 1/1,000 in PBS-T 2% BSA (working concentration, 0.4 mg/ml), which had been covalently tagged with fluorochromes Alexa 546 and Alexa 647. Finally, the slides were washed 2 times with PBS-T, dried, and scanned with a ScanArray Express (PerkinElmer, Waltham, Mass., USA).

### Statistical Analysis

Microarray analysis was performed according to the method of Lin and colleagues (14). This method is based on the calculation of a Z-score, which is a measure of the standardized fluorescent intensity for IgE and IgG4 recognition. In brief, the Z-score was calculated for each peptide spot (Z_i_) using the PPS values within the same array according to the formula: Zi = (S_i_-S_PBS_)/MAD(S_PBS_), where S for each peptide spot (Si) or PPB (S_PBS_) is the median fluorescence signal of the spot divided by local background and log_2_ transformed. MAD(S_PBS_) is the median absolute deviation of all the read-outs of PBS spots. The total Z-value for each peptide is the median of the Z-scores of the three replicate spots within the same microarray and denotes the average of standardized intensities of fluorescence (ch1 IgG4-Alexa 647 and ch2 IgE-Alexa 546). An individual peptide sample was considered positive if the average Z-score exceeded 3 after subtracting the average Z-score controls for each peptide. Data analysis and presentation were performed using Microsoft Excel, Sigmaplot (Systat Software, Inc., San Jose, Calif., USA), and TIGR MultiExperiment Viewer (Mev v3.1) software.

For the statistical analysis of epitope recognition, we performed the one-sample Student *t*-test to reject the null hypothesis that the ratio of IgE to IgG4 standardized binding intensities equals zero. All of these tests were done with Mev v3.1 software. We did not use any false discovery correction, and *p* < 0.05 was considered statistically significant.

For clinical and demographic patient data, quantitative variables are shown as means and standard deviation for normal distributions or median and interquartile ranges for nonnormal distributions. Qualitative variables are presented as frequencies (percentages). Normality was assessed by Shapiro–Wilk test. Means between groups when normality was followed were compared with the Student *t*-test, whereas medians between groups were compared using the Mann-Whitney test. χ^2^ test (or the Fisher exact test, when needed) was used to compare proportions. The data were analyzed with statistical software Stata/IC 12.0. Significance was set at *p* < 0.05.

## Results

### Patient Clinical Characteristics

A total of 46 peach-allergic patients sensitized to peanuts were enrolled. A total of 35 patients were allergic to peanuts according to a clinical IgE-mediated recent history of reactions and positive SPT response (peanut-allergic group). Peanut ingestion caused symptoms in the 35 peanut-allergic patients: nine patients had oral allergy syndrome (OAS), 15 had non-anaphylactic systemic symptoms, and 11 had anaphylaxis. A total of 11 peanut-tolerant patients had positive peanut SPT responses with no symptoms of peanut intake, as confirmed by an open oral challenge (peanut-tolerant group). The median age of the peanut-allergic patients was 29 (15–48) years and 28 (18–45) years for peanut-tolerant patients and the majority were female (77% in the peanut-allergic group and 73% in the peanut-tolerant group). No demographic differences were observed between the peanut-allergic and peanut-tolerant groups (Table 1).

**Table 1:** Patients characteristics.

	**Peanut allergic group**	**Peanut tolerant group**	***P***
Number of patients	35	11	-
Sex, *n* (%)			
Female	27 (77%)	8 (73%)	0.765
Age, years, mean (maximum, minimum)	29 (15–48)	28 (18–45)	0.642
Peach symptoms, % (*n*)			**0.048**
OAS	34% (12)	18.2 % (2)	
Systemic symptoms	49% (17)	27.3% (3)	
Anaphylaxis	17% (6)	54.5% (6)	
Peanut symptoms, % (*n*)			-
OAS	26% (9)	0 (0)	
Systemic symptoms	43% (15)	0 (0)	
Anaphylaxis	31% (11)	0 (0)	
Specific IgE, kU_A_/L, median (IQR)			
Peanut	2.14 (0.42–5.36)	1.12 (0.47–12.5)	0.673
Peach	5.6 (3.75–19.1)	6.61(2.23–30.55)	0.919
Ara h 9	3.43 (1.43–11.8)	4.85 (1.49–24.1)	0.495
Pru p 3	6 (3.46–17.8)	7.43 (2.53–34.5)	0.738
Specific IgG_4_, kU_A_/L, median (IQR)			
Ara h 9	0.1 (0.03–0.45)	0.43 (0.23–1.18)	**0.039**
Pru p 3	0.39 (0.14–1.13)	0.92 (0.39–4.65)	**0.039**
Specific IgG_4_/IgE, median (IQR)			
Ara h 9	0.03 (0.01–0.08)	0.09 (0.05–0.27)	**0.034**
Pru p 3	0.05 (0.02–0.13)	0.13 (0.06–0.35)	**0.047**

*Bold values indicate statistically significant differences (p < 0.05)*.

Peach ingestion caused OAS in 12 peanut-allergic patients, non-anaphylactic systemic symptoms (e.g., urticaria, gastrointestinal symptoms) in 17 peanut-allergic patients, and anaphylaxis in six peanut-allergic patients. Peach ingestion triggered OAS in two patients, systemic symptoms in three patients, and anaphylaxis in six peanut-tolerant patients. Differences in the severity of symptoms after eating peach were detected between peanut-allergic and peanut-tolerant patients since anaphylaxis was more frequent in peanut-tolerant patients (54.5%) than in peanut-allergic patients (17%). Demographic and clinical data from peanut-allergic and peanut-tolerant patients are summarized in Table 1.

### sIgE/IgG4 Levels to Peanut/Peach Extract and Peanut/Peach Components

No differences in sIgE levels against peanut or peach were observed between the peanut-allergic and peanut-tolerant groups (Table 1). Serum sIgE levels to Ara h 9 and Pru p 3 were also similar between both groups (Table 1). sIgE against rAra h 1, rAra h 2, rAra h 3, and rAra h 8 were negative for all patients; thus, additional sensitization apart from LTPs was discarded.

In contrast, peanut-tolerant patients showed higher sIgG4 levels to Ara h 9 and Pru p 3 than peanut-allergic subjects; consequently, the IgG4/IgE ratio of these patients was significantly higher (Table 1).

### IgE and IgG4 Antibody-Binding to Sequential Epitopes of Ara h 9 and Pru p 3

We examined the IgE and IgG4 binding to sequential epitopes using a peptide microarray corresponding to linear sequences of the LTPs Ara h 9 and Pru p 3, the major peanut and peach allergens in the Mediterranean area. Overall, independently of their phenotype, the percentage of peptides recognized by IgE was similar in both proteins, 39.2%. On the other hand, the percentage of peptides recognized by IgG4 was much smaller than IgE, with 2.5% of Ara h 9 and 5.8% of Pru p 3.

Comparing the frequency of IgE- and IgG4-binding epitopes of the peanut-allergic group and peanut-tolerant group, we found a substantial increase in IgE recognition in the peanut-tolerant group for both LTPs. The percentage of IgE-positive peptides of Ara h 9 in peanut-allergic patients was 34.3%, while in peanut-tolerant patients it was 60.4% (*p* = 0.0009). Similarly, the percentage of IgE-positive peptides of Pru p 3 in peanut-allergic patients was 32.3 and 65.8% in peanut-tolerant patients (*p* < 0.0001). The percentage of IgG4-positive peptides was also higher in peanut-tolerant patients than in peanut-allergic patients for both LTPs (Ara h 9: 1.6% in allergic patients and 5.5% in tolerant patients, *p* = 0.001; Pru p 3: 4.2% in allergic patients and 10.5% in tolerant patients, *p* = 0.0006).

**Figure 1:**
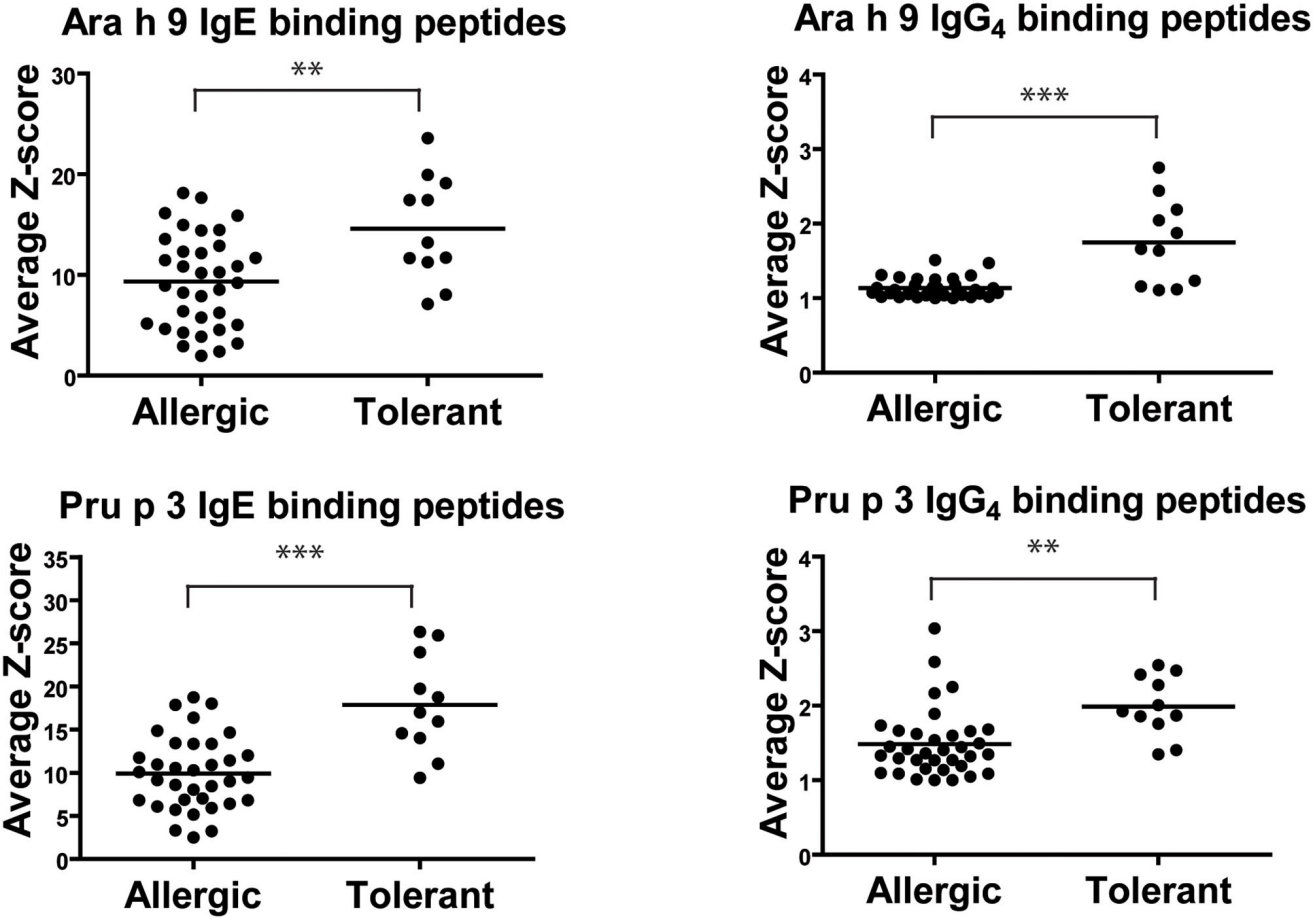
Average IgE and IgG4 peptide binding to overlapping peptides corresponding to a linear sequence of Ara h 9 and Pru p 3. Each data point represents the average intensity of peptide recognition for each patient calculated as average *Z*-scores. The horizontal black line represents the mean. Statistical analysis was performed by Student *t*-test. Asterisks indicate statistically significant differences between peanut-allergic and peanut-tolerant patients (**p < 0.01 and ***p < 0.001).

The intensity of IgE recognition for each peptide of both LTPs in peanut-tolerant patients was higher than in peanut-allergic patients, as represented by the average intensity of fluorescence or Z-score (Figure 1). The average intensity (average Z-score) of IgE recognition of Ara h 9 peptide in peanut-tolerant patients was 14.6 ± 1.6, while in peanut-allergic patients it was 9.3 ± 0.8 (*p* = 0.003). The average intensity (average Z-score) of IgE recognition of Pru p 3 average in peanut-tolerant patients was 17.9 ± 1.7 and 9.9 ± 0.7 in peanut-allergic patients (*p* < 0.0001). We also found significant differences in the intensity of IgG4 recognition of both Ara h 9 and Pru p 3 between both groups. The average intensity of IgG4 recognition of Ara h 9 in peanut-tolerant patients was 1.8 ± 0.2 and 1.1 ± 0.02 in peanut-allergic patients (*p* < 0.0001). The average intensity of IgG4 recognition of Pru p 3 was also significantly higher in peanut-tolerant patients than in peanut-allergic patients (2 ± 0.1 and 1.5 ± 0.08 respectively, *p* = 0.002).

**Figure 2:**
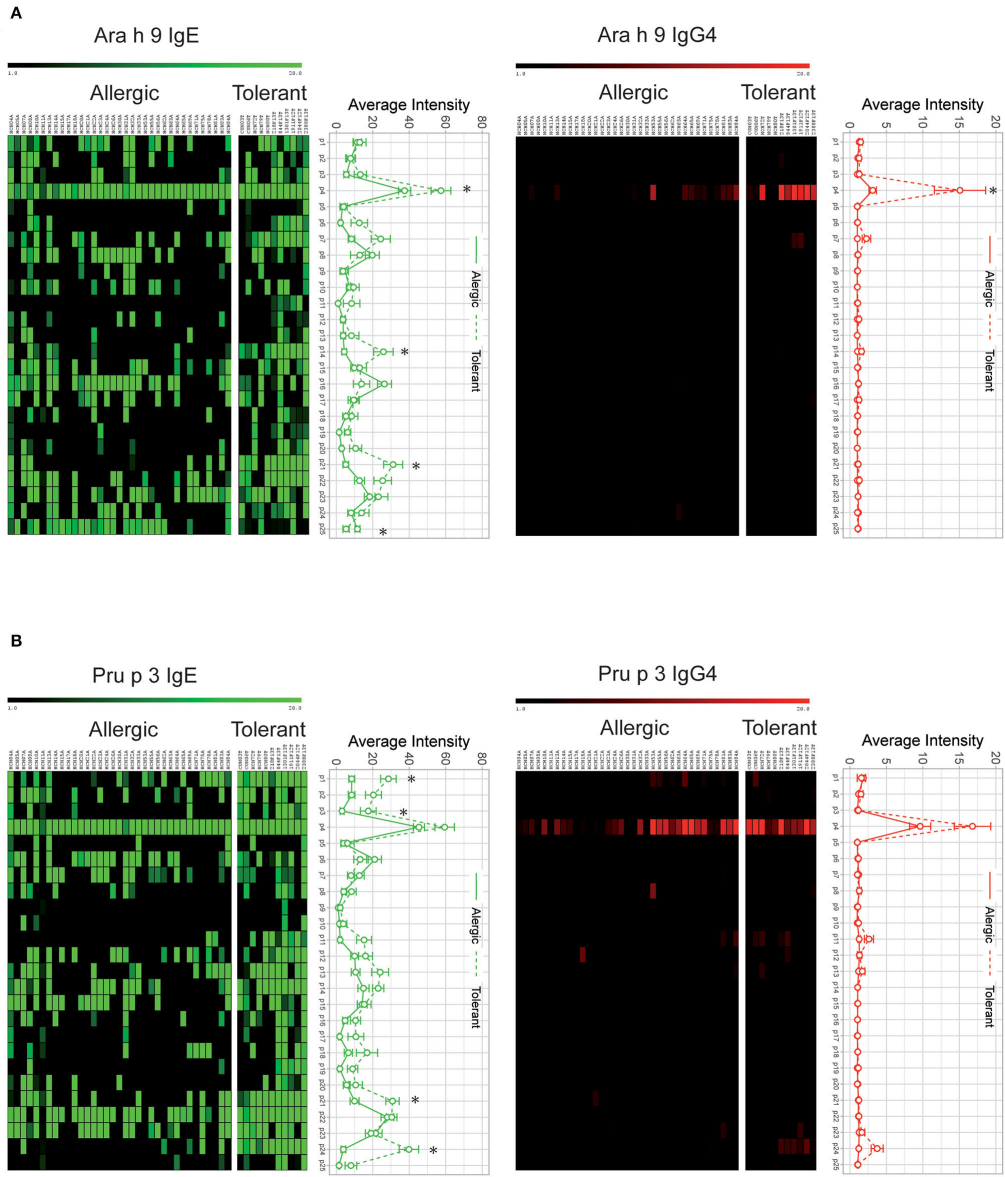
IgE and IgG4 peptide binding to overlapping peptides corresponding to a linear sequence of Ara h 9 **(A)** and Pru p 3 **(B)**. The intensity of IgG4 and IgE binding is represented by average *Z*-scores on a grading scale according to the scale bar at the top of the figure. Individual sera of allergic and tolerant patients are shown in the columns and 20-aa peptide sets are shown in the rows. Statistical analysis was performed by Student t test. Asterisks indicate statistically significant differences between peanut-allergic and peanut-tolerant patients (*p* < 0.05). Error bars represent standard error (SE).

Analyzing each individual peptide, we found four Ara h 9 peptides (p4, p14, p21, and p25) and four Pru p 3 peptides (p1, p3, p21, and p24) with a significantly elevated IgE recognition in peanut-tolerant patients compared with peanut-allergic patients (Figure 2). Similarly, the intensity of IgG4-peptide recognition was higher in peanut-tolerant patients, although with fewer recognized peptides (Figure 2). Interestingly, only peptide 4 of Ara h 9 was found to be elevated in peanut-tolerant patients, with significant differences between both groups of patients (Figure 2).

**Figure 3:**
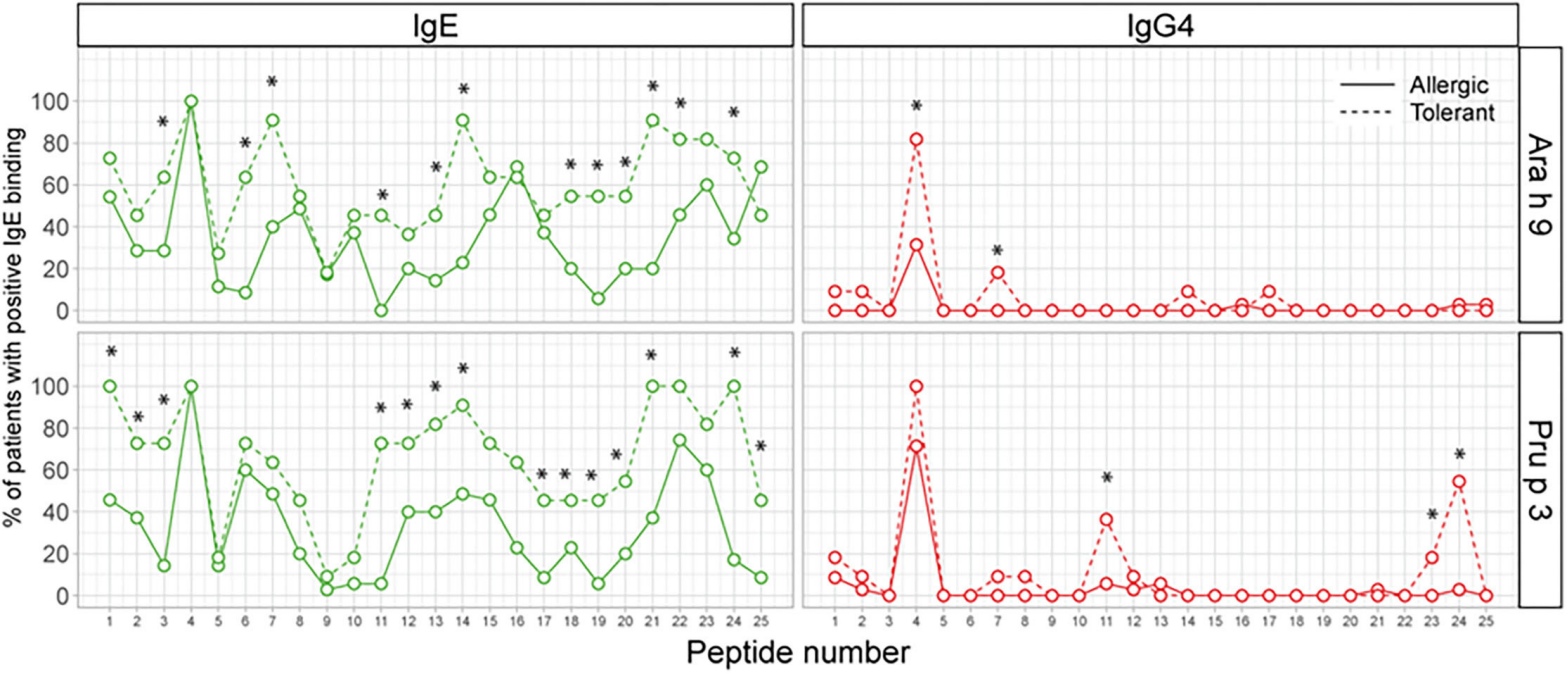
Percentage of patients with positive recognition for each peptide of Ara h 9 and Pru p 3. The X-axis shows the number of overlapping peptides on the microarray. The Y-axis shows the percentage of patients within each group who showed positive binding (*Z*-score >3). Statistical analysis was performed by χ^2^ test. Asterisks indicate statistically significant differences between peanut-allergic and peanut-tolerant patients (*p* < 0.05).

Next, we analyzed the percentage of patients with positive recognition for each peptide of both LTPs. As shown in Figure 3, a large number of peptides either of Ara h 9 and Pru p 3 were recognized more frequently by IgE in peanut-tolerant—patients than in peanut-allergic patients. On the other hand, only two Ara h 9 peptides (one of which was peptide 4) and three Pru p 3 peptides were recognized more frequently by IgG4 in peanut-tolerant patients than in peanut-allergic patients.

### Ara h 9 and Pru p 3 Peptide 4

**Figure 4:**
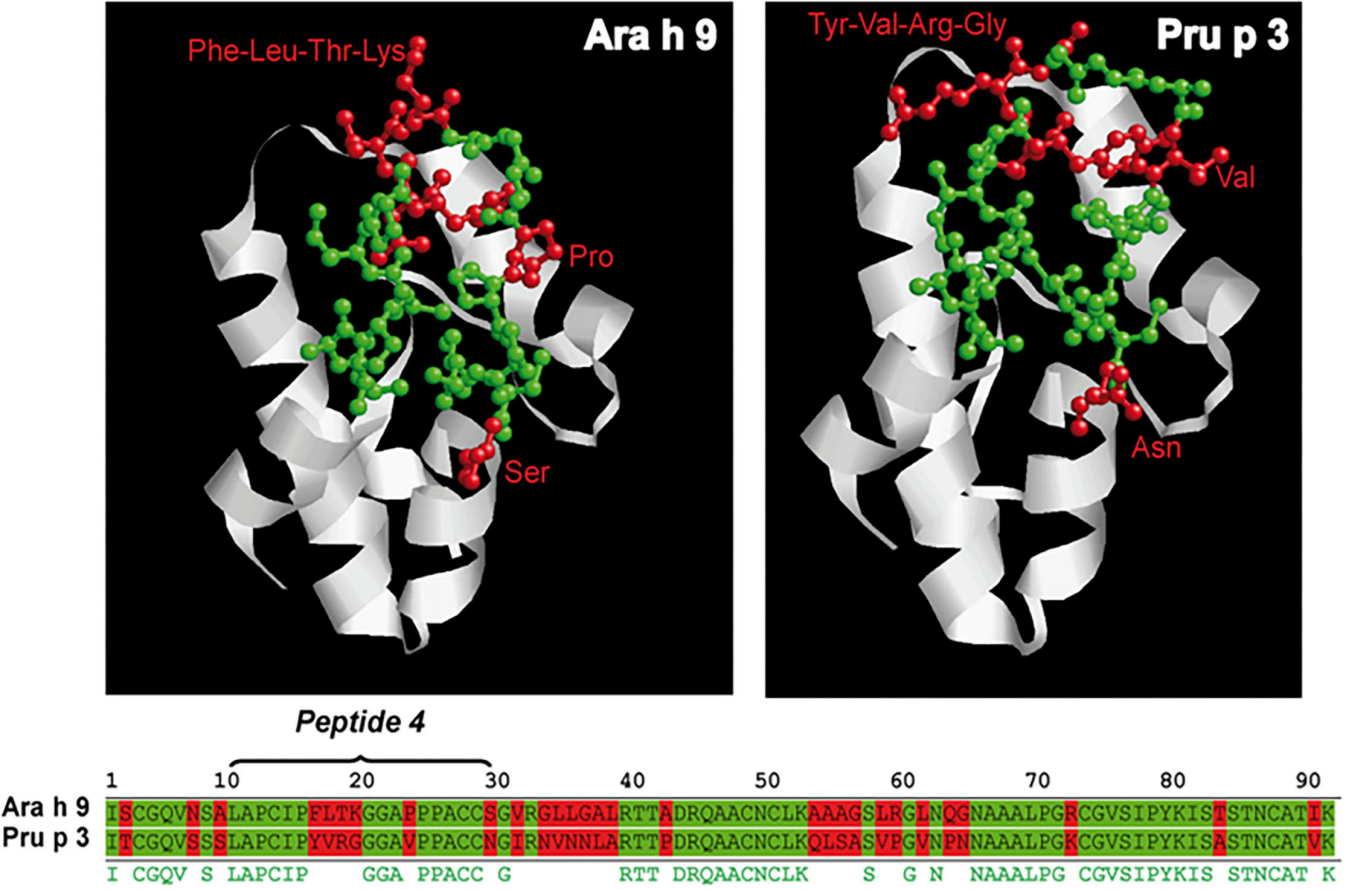
Sequence and ribbon diagrams of Ara h 9 and Pru p 3 used as model to represent peptide 4 location of each protein (conserved aa are highlighted in green and non-conserved aa are highlighted in red).

Given the association of IgG4 binding to peptide 4 of Ara h 9 with peanut tolerance, we compared the sequence of peptide 4 of Ara h 9 with peptide 4 of Pru p 3 and we localized peptide 4 onto the published structure of both proteins. Peptide 4 of Ara h 9 differed in seven amino acids from peptide 4 of Pru p 3 (Figure 4). In both LTPs, four consecutive amino acids (Ara h 9 FLTK and Pru p 3 YVRG) in the center of peptide 4 located at the end of an alpha helix exposed to the protein's surface could explain the different recognition between the two proteins.

All peach-allergic patients, regardless of whether they were peanut-tolerant or peanut-allergic, recognized peptide 4 by IgE in Ara h 9 and Pru p 3. However, IgE Ara h 9 peptide 4 recognition intensity was higher in peanut-tolerant than peanut-allergic subjects. This difference in IgE-intensity binding between peanut-allergic and peanut-tolerant patients was not observed for peptide 4 Pru p 3.

**Figure 5:**
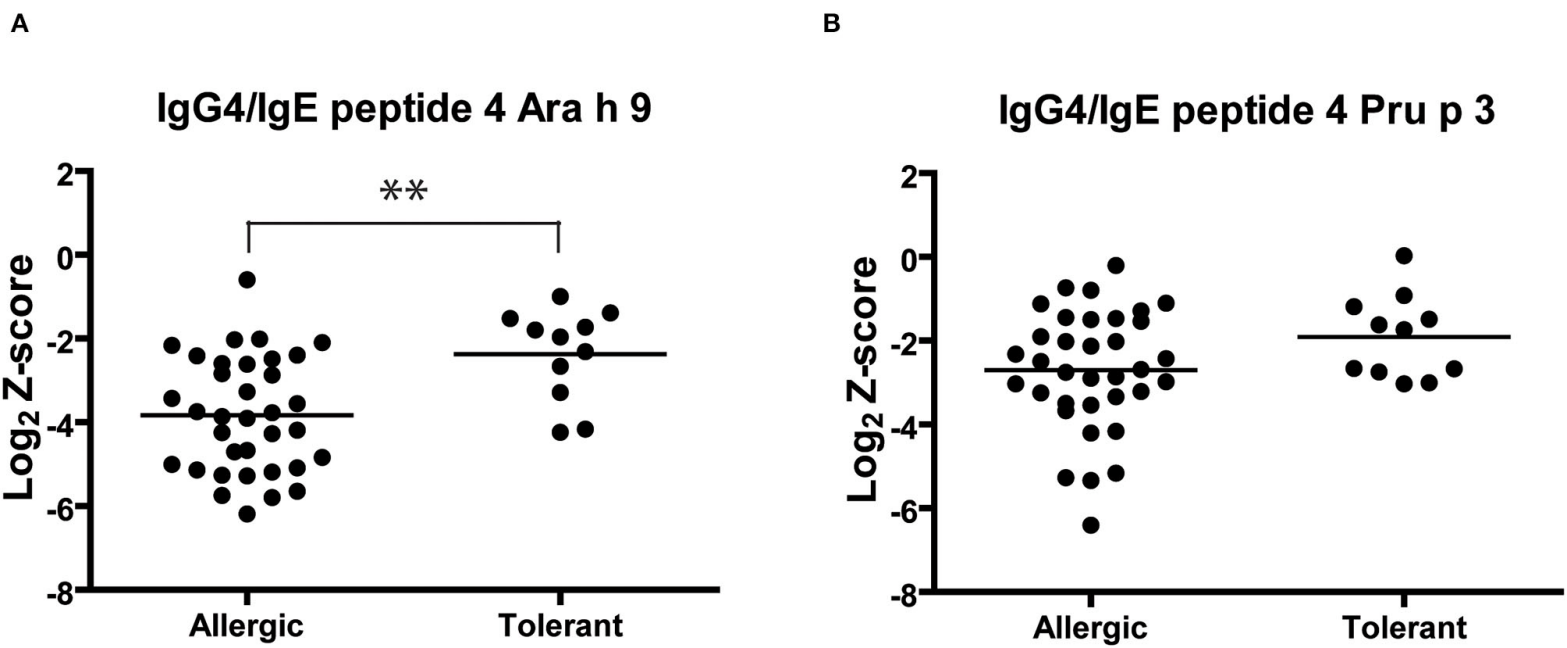
IgG4/IgE ratio of Ara h 9 **(A)** and Pru p 3 **(B)** peptide 4 recognition in peanut-allergic and peanut-tolerant patients. Statistical analysis was performed by Student t test. Asterisks indicate statistically significant differences between peanut-allergic and peanut-tolerant patients (**p < 0.01).

Finally, we analyzed the IgG4/IgE ratio of recognition of Ara h 9 and Pru p 3 peptide 4 (Figure 5). The IgG4/IgE ratio of Ara h 9 peptide 4 was significantly higher in peanut-tolerant than peanut-allergic patients, while no significant differences were observed in Pru p 3. Consequently, an increase in the IgG4 recognition of Ara h 9 peptide 4 could be associated with peanut tolerance.

## Discussion

Food allergy is difficult to predict in polysensitized patients, since cross-reactivity is only expressed serologically, but does not lead to clinically relevant symptomatology. This pitfall is clearly expressed in patients showing LTP sensitization, since a broad sensitization to plant foods can be determining the clinical relevance of Ara h 9, but is not always related to the clinical expression (8, 15, 16). Peach allergy is the most common allergy in LTP sensitization (7, 8, 17) and peach LTP Pru p 3 sensitization seems to be an initiator allergen (5, 17).

Predominant sensitization to Ara h 1-3 allergens is seen in peanut-allergic patients in non-Mediterranean areas as well as in allergic children in Mediterranean areas (18). However, characteristically, non-pediatric peanut-allergic patients from the Mediterranean area are mainly sensitized to Ara h 9 and frequently have associated peach allergy (2, 4, 5, 19). This Mediterranean pattern has also been recently reported in non-Mediterranean areas (9, 20–22). Peach LTP-specific and peanut LTP-specific IgE are very often associated, with high cross-reactivity between both molecules reported (4). Thus, the clinical relevance of Ara h 9 sensitization in peach-allergic patients is a clinical challenge, since overlapping values of specific IgE to peanut/Ara h 9 between peanut-tolerant and peanut-allergic patients showing LTP sensitization have been observed (2, 5, 23). Moreover, the clinical expression of this sensitization can change over time, since one-third of LTP-sensitized patients will develop new food allergies in the long term (24, 25). Until now, there are no validated biomarkers to predict peanut allergy in peach-allergic patients with LTP sensitization.

In the current study, we identified for the first time, using peptide microarray technology, IgE and IgG4 sequential epitopes of Pru p 3 and Ara h 9 in peach-allergic patients sensitized to peanuts. We found significant differences in IgE and IgG4 binding between patients who tolerate peanuts and those who present an allergy to peanuts. Interestingly, only peptide 4 of Ara h 9 was found to be significantly elevated in IgG4 recognition in peanut-tolerant patients.

Other studies have also observed food tolerance related to polyclonal IgE binding. Otsu and colleagues found that patients with relatively severe allergic responses to peanut exposure recognized fewer IgE linear epitopes of both Ara h 2 and Ara h 6 than subjects with less severe responses to peanuts (26). Although these results must be taken with caution since other authors have found more diverse IgE peptide recognition against Ara h 1 and Ara h 2 in patients with more severe allergic reactions to peanuts (27). Furthermore, the peanut-specific IgE and the intensity of binding of IgE against Ara h 2 and Ara h 6 had no discernible relationship with the severity of the reactions to peanuts (26). The similar polyclonal expansion has been observed in some patients after oral peanut immunotherapy (28, 29) suggesting that new epitope-specific B-cell clones are sorted even when peanut IgE response is suppressed.

It is important to mention that our food allergy model is quite different from previous food allergy scenarios where IgE/IgG4 is measured against peptides in spontaneously peanut tolerant patients (26) and in induced peanut tolerant patients (28, 29). However, our patients (>14 years old) suffer from peach allergy through the LTP Pru p 3 and peanut LTP Ara h 9 sensitization is caused by cross reactivity. At the moment of the study, peanut tolerant patients showed serological cross reactivity between Ara h 9 and Pru p 3 but no clinical cross reactivity. We can hypothesize that, in our study, peanut tolerance could be related to a lack of IgE-nonrelated sensitization of the main initiator of LTP syndrome Pru p 3. Even more, it has been reported (24, 25) that one-third of the patients react to new plant food along the natural course of this allergy, suggesting a switch in previous non-clinical Ara h 9 sensitization to clinically relevant sensitization could happen. It would be interesting to re-evaluate our patients after a few years (avoiding sublingual peach immunotherapy), checking if previous peanut tolerant patients developed peanut allergy and correlating data with IgE/IgG4 peptide binding findings.

Tolerance is also associated with a wider IgG4 repertoire (29). In our study, IgG4-detected peptides of Ara h 9 and Pru p 3 were more frequently observed in peanut-tolerant patients than in peanut-allergic patients, but only in 2 and 3 peptides, respectively. Nevertheless, our peach-allergic patients showed tolerance to peanuts in a primary way, different from induction of tolerance by oral immunotherapy.

It is also interesting to note that, together with Ara h 9-specific IgG4 levels, Pru p 3-IgG4 levels were also higher in peanut-tolerant patients than in peanut-allergic patients (all of whom were peach-allergic). In addition, a different IgE recognition pattern of Pru p 3 was observed in peanut-allergic patients compared with peanut-tolerant patients. These findings suggest that the way in which Pru p 3 is recognized by the immune system could determine the clinical expression pattern of IgE-derived cross-reactivity in LTP sensitization. This concept merits further study in order to potentially identify a biomarker to predict the evolution from a mono-LTP-sensitized peach-allergic patient to the complex LTP syndrome expressed by multiple allergies to plant foods and pollen.

The differences observed in IgE-peptide recognition of Ara h 9 or Pru p 3 in our study cannot explain the clinical phenotype of peach-allergic patients sensitized to peanuts. However, IgG4 recognition of peptide 4 of Ara h 9 was significantly higher in peanut-tolerant patients compared with peanut-allergic patients. On the other hand, all patients exhibited high-intensity recognition of peptide 4 of Pru p 3 by both IgE and IgG4, which is consistent with the fact that all of the patients were allergic to peach. These data suggest that IgG4 antibodies against Ara h 9 peptide 4 could be associated with peanut tolerance in peach-allergic patients.

The Pru p 3 IgE epitopes described herein are consistent with those described by García-Casado and colleagues using Spot technology (30). They described three major IgE-binding regions of the Pru p 3 (positions 11–25, 31–45, and 71–85). The first and third regions match perfectly with the epitopes described in our study (peptides 4 and 22), and the second region partially matches (peptides 7, 8, 11, and 12) (Supplementary Figure 1). It is important to mention that in García-Casado's study, the second region was not recognized by all patients, which could explain the differences in our results. On the other hand, the first region showed the most prominent recognition profile, which matched with our peptide 4. In our study, all peach-allergic patients showed recognition of peptide 4 of Ara h 9, however, peanut-tolerant patients showed higher IgE-binding intensity to this epitope. This fact, together with the different roles of IgG4 antibodies, may be explained by sequence differences between peptide 4 of both proteins, with sequence differences in the peptide center zone. This area has been shown to be essential for IgE recognition, and the change of three amino acids in this region fully abolishes the IgE recognition by peach allergic patient sera (30). These results would be consistent with the blocking role of IgG4 previously described in a generic way (31) and more specifically in peanut-allergic patients (32).

Apart from the short sample, a limitation of this study is that the IgE/IgG4 binding affinity has not been studied. However, pleiotropic Ara h 9 IgE binding has been observed in tolerant peanut patients compared with peanut allergic patients, and specific IgG4 recognition of Ara h 9 peptide 4 has been observed in the wide peptide array. Differences observed in peanut allergic and tolerant patients suggest that Ara h 9 peptide 4-IgG4 could oust IgE, inducing tolerance. Plant food LTP IgE affinity should be deeply studied in LTP syndrome progression.

In this sense, epitope recognition by IgE and IgG4 appears to be attractive biomarkers to better understand the gap between immunoglobulin-specific allergen binding and clinical expression. Changes in linear epitope recognition for some peanut allergens, Ara h 1-3 and Ara h 6 have been characterized and associated with peanut allergy severity (26, 27) or clinical course prediction in children (33, 34). Linear (30) and conformational epitopes (30, 35) have been described as the main sensitizer in LTP-allergic patients, Pru p 3. Moreover, both linear and conformational epitopes seem to be responsible for cross-reactivity between Pru p 3 and Tri a 14 LTPs (36). Since Pru p 3 is a very stable allergen (37), linear epitopes could be responsible for cross-reactivity between LTPs, such as Pru p 3 and Ara h 9. The clinical relevance of this epitope recognition has been analyzed in this paper for the first time. In contrast to foods such as milk, the clinical significance of recognizing the linear epitopes of the different LTPs and their cross-reactivity is unknown.

The clinical relevance of this epitope recognition has been analyzed in this study for the first time. In contrast to foods such as milk, the clinical significance of recognizing the linear epitopes of the different LTPs and their cross-reactivity is unknown.

Therefore, as previously proposed and consistent with our results, differences in sIgE levels or IgE-binding peptides are not correlated to the clinical phenotype of patients with peanut allergy, while the blocking IgG4 antibodies could provide an additional explanation for the absence of clinical reactivity (32). More studies are required to confirm this observation in a wider sample and other sensitizations in the complex LTP syndrome.

## Data Availability Statement

The data supporting the findings of this study are presented within the article or as Supplementary Materials. Further inquiries may be directed to the corresponding author.

## Ethics Statement

The studies involving human participants were reviewed and approved by (032/2009 and 3/9/2009) University of Navarra. Written informed consent to participate in this study was provided by the participants' legal guardian/next of kin.

## Author Contributions

MG conceived the original idea, enrolled patients and wrote the manuscript. FG recruited patients and supervised the manuscript. MF enrolled patients and supervised the manuscript. CF-L performed the experiments and analyzed data. LS-R performed the experiments and analyzed data. JM-B designed experiments, analyzed data and wrote the manuscript. BH conceived the original idea, designed experiments and wrote the manuscript. All authors contributed to the article and approved the submitted version.

## Funding

This work was supported by the Institute of Health Carlos III (ISCIII) (PS09/01083) and of the Ministry of Economy and Competitiveness (PI17/01318), co-founded by the European Regional Development Fund (ERDF) for ARADyAL Thematic Networks and Cooperative Research Centers (RD1600060031; RD16/0006/0001; RD16/0006/0009) and Senior Clinical Researcher Program (B-0005-2019).

## Conflict of Interest

The authors declare that the research was conducted in the absence of any commercial or financial relationships that could be construed as a potential conflict of interest.

## Publisher's Note

All claims expressed in this article are solely those of the authors and do not necessarily represent those of their affiliated organizations, or those of the publisher, the editors and the reviewers. Any product that may be evaluated in this article, or claim that may be made by its manufacturer, is not guaranteed or endorsed by the publisher.

## Supplementary Material

Supplementary Figure 1 IgE peptide binding to overlapping peptides corresponding to a linear sequence of Ara h 9 and Pru p 3. The X-axis shows the number of overlapping peptides on the microarray. The Y-axis shows the average IgE binding represented as weighted average Z-scores. Previously reported epitopes reported by García-Casado and colleagues (30) are shown in the box.
