# Supplementary figures and images for: Differences in Linear Epitopes of Ara h 9 Recognition in Peanut Allergic and Tolerant, Peach Allergic Patients
Source: Front Allergy. 2022 Jul 22;3:896617. doi: 10.3389/falgy.2022.896617 (PMC9352880; doi:10.3389/falgy.2022.896617)

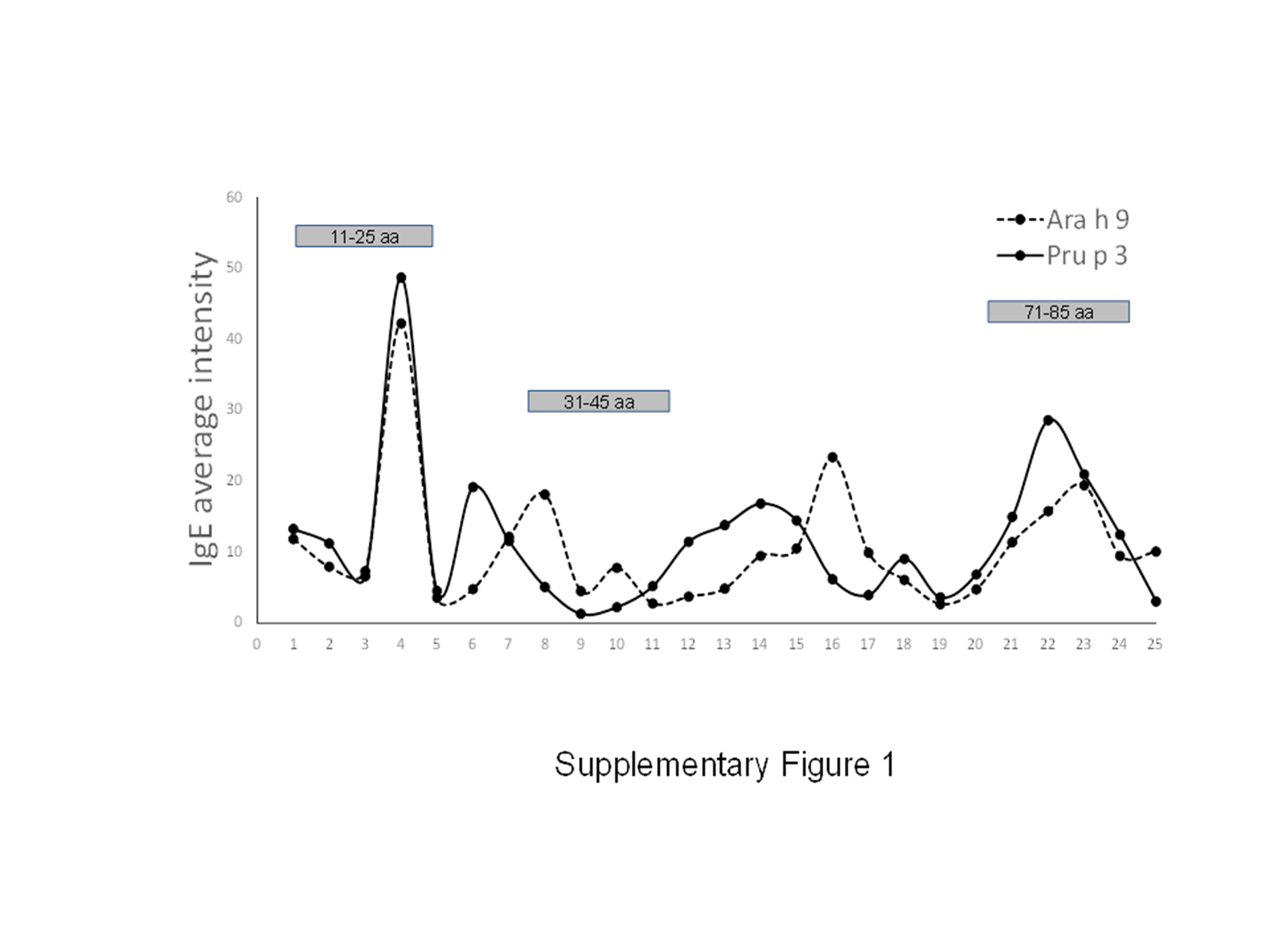

Supplement: Supplementary Figure 1 — IgE peptide binding to overlapping peptides corresponding to a linear sequence of Ara h 9 and Pru p 3. The X-axis shows the number of overlapping peptides on the microarray. The Y-axis shows the average IgE binding represented as weighted average Z-scores. Previously reported epitopes reported by García-Casado and colleagues (30) are shown in the box. [file Image_1.tif]
